# An evaluation of the emerging interventions against Respiratory Syncytial Virus (RSV)-associated acute lower respiratory infections in children

**DOI:** 10.1186/1471-2458-11-S3-S30

**Published:** 2011-04-13

**Authors:** Harish Nair, Vasundhara R Verma, Evropi Theodoratou, Lina Zgaga, Tanvir Huda, Eric AF Simões, Peter F Wright, Igor Rudan, Harry Campbell

**Affiliations:** 1 Centre for Population Health Sciences, Global Health Academy, The University of Edinburgh, UK; 2 Public Health Foundation of India, New Delhi, India; 3 International Centre for Diarrhoeal Disease Research, Bangladesh (ICDDR,B), Dhaka, Bangladesh; 4 University of Colorado Denver and The Children’s Hospital, Denver, CO, USA; 5 Division of Infectious Disease and International Health, Dartmouth Medical School, Lebanon, NH, USA; 6 Croatian Centre for Global Health, University of Split Medical School, Croatia

## Abstract

**Background:**

Respiratory Syncytial Virus (RSV) is the leading cause of acute lower respiratory infections (ALRI) in children. It is estimated to cause approximately 33.8 million new episodes of ALRI in children annually, 96% of these occurring in developing countries. It is also estimated to result in about 53,000 to 199,000 deaths annually in young children. Currently there are several vaccine and immunoprophylaxis candidates against RSV in the developmental phase targeting active and passive immunization.

**Methods:**

We used a modified CHNRI methodology for setting priorities in health research investments. This was done in two stages. In Stage I, we systematically reviewed the literature related to emerging vaccines against RSV relevant to 12 criteria of interest. In Stage II, we conducted an expert opinion exercise by inviting 20 experts (leading basic scientists, international public health researchers, international policy makers and representatives of pharmaceutical companies). The policy makers and industry representatives accepted our invitation on the condition of anonymity, due to the sensitive nature of their involvement in such exercises. They answered questions from the CHNRI framework and their “collective optimism” towards each criterion was documented on a scale from 0 to 100%.

**Results:**

In the case of candidate vaccines for active immunization of infants against RSV, the experts expressed very low levels of optimism for low product cost, affordability and low cost of development; moderate levels of optimism regarding the criteria of answerability, likelihood of efficacy, deliverability, sustainability and acceptance to end users for the interventions; and high levels of optimism regarding impact on equity and acceptance to health workers. While considering the candidate vaccines targeting pregnant women, the panel expressed low levels of optimism for low product cost, affordability, answerability and low development cost; moderate levels of optimism for likelihood of efficacy, deliverability, sustainability and impact on equity; high levels of optimism regarding acceptance to end users and health workers. The group also evaluated immunoprophylaxis against RSV using monoclonal antibodies and expressed no optimism towards low product cost; very low levels of optimism regarding deliverability, affordability, sustainability, low implementation cost and impact on equity; moderate levels of optimism against the criteria of answerability, likelihood of efficacy, acceptance to end-users and health workers; and high levels of optimism regarding low development cost. They felt that either of these vaccines would have a high impact on reducing burden of childhood ALRI due to RSV and reduce the overall childhood ALRI burden by a maximum of about 10%.

**Conclusion:**

Although monoclonal antibodies have proven to be effective in providing protection to high-risk infants, their introduction in resource poor settings might be limited by high cost associated with them. Candidate vaccines for active immunization of infants against RSV hold greatest promise. Introduction of a low cost vaccine against RSV would reduce the inequitable distribution of burden due to childhood ALRI and will most likely have a high impact on morbidity and mortality due to severe ALRI.

## Background

Respiratory Syncytial Virus (RSV) is the commonest cause of acute lower respiratory tract infections (ALRI), here defined as pneumonia and bronchiolitis, in children under the age of 5 years (22% of all ALRI episodes) and is estimated to be responsible for about 53,000 to 199,000 deaths annually [1]. A majority of the episodes of RSV-associated ALRI in young children occur in the first year of life. Stang estimated that the annual economic burden due to RSV-LRI hospitalisation in the United States alone is $43.2 to $69.1 million for all children aged less than 5 years and $36.5 to $58.5 million in the case of infants [2]. RSV is thought to account for approximately 85% of cases of bronchiolitis and approximately 20% of cases of childhood pneumonia [3]. Though in most cases the infection resolves without any sequelae, in some cases it can impact on the future health state of the child. Several studies have demonstrated an association between RSV infection in the first two years of life and the subsequent development of wheezing and LRI hospitalisations in the first decade of life [4-7]

Presently, there is no effective vaccine to combat this significant disease burden. Several candidate vaccines as well as immunoprophylaxis which hold promise are under various stages of development. We aimed to review the existing literature, outlining the progress of the emerging vaccines and immunoprophylaxis against RSV at all stages of development; present the evidence regarding key issues surrounding these products and assess the level of collective optimism of international experts over its priority status for receiving investment support. The paper is presented as part of a series of papers, each in turn focusing on different emerging vaccines and other interventions against pneumonia.

## Methods

We used a modified Child Health and Nutrition Research Initiative (CHNRI) methodology for setting priorities in health research investments. The methodology has been described in great detail [8-12] and implemented in a variety of settings [13-18].

### CHNRI exercise – stage I: identification and selection of studies

**Figure 1:**
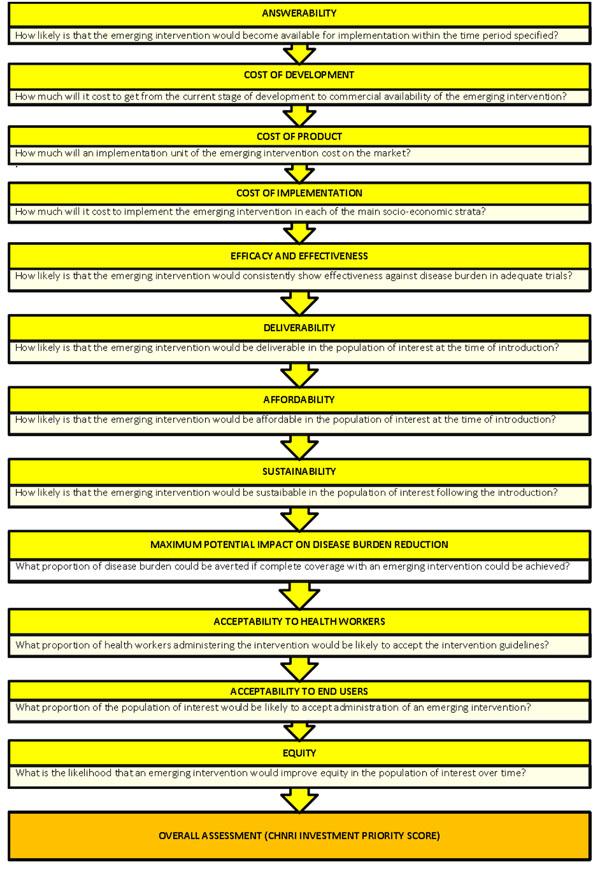
**A summary of Stage I of the CHNRI process of evaluation of an emerging intervention (a systematic review of the key CHNRI criteria).** CHNRI- Child Health and Nutrition Research Initiative

We conducted a systematic literature review using the following criteria: answerability, cost of development, cost of product, cost of implementation, efficacy and effectiveness, deliverability, affordability, sustainability, maximum potential impact on disease burden reduction, acceptability to health workers, acceptability to end users and equity [15] (Figure 1). The following search terms: respiratory syncytial virus, vaccination, immunization, infants, and children were used. Specific terms were used for active and maternal immunization and for the specific criteria using MeSH headings and truncation (Supplementary table S1 in additional file 1). The search was limited to Ovid MEDLINE, Embase, Global Health, Web of Science, LILACS, IndMed, and grey literature (SIGLE) databases from January 1994 to July 2009 (updated in August 2010). This was supplemented with hand searching of online journals and scanning of reference lists of identified citations. A total of 3138 articles were identified initially of which 70 articles were found suitable for full-text review. The inclusion and exclusion criteria are outlined in Table 1.

**Table 1:** Details of eligibility criteria used for screening the studies

Inclusion criteria	Exclusion Criteria
**- Included research into RSV vaccine, or other vaccine that may bear resemblance to future RSV vaccination programs**	- RSV vaccine candidate was not a focus of the paper
**- Vaccine research was targeted at children under 5 years**	- Bovine RSV vaccine or vaccine for the elderly
**- Gave an indication of answerability, efficacy, effectiveness, delivery, disease burden reduction or impact on equity of a vaccine**	- Papers not directly relating to vaccine development and its impact

### CHNRI exercise – stage II: an expert opinion exercise

**Figure 2:**
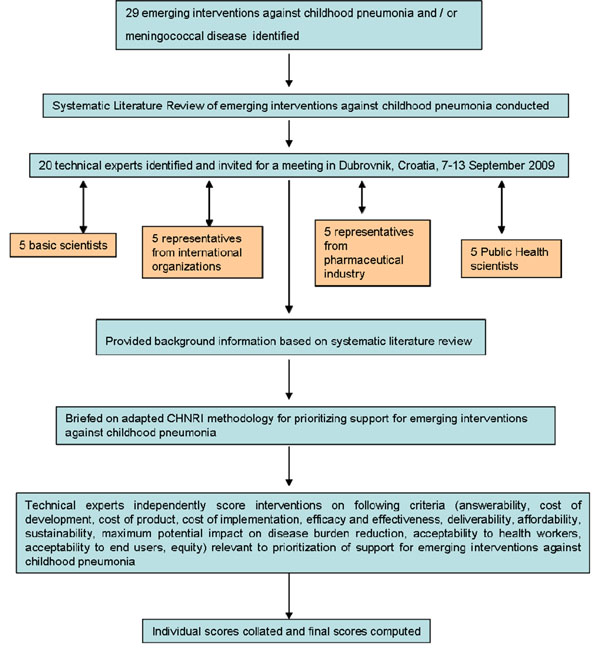
**A summary of Stage II of the CHNRI process of evaluation of an emerging intervention (an expert opinion exercise using the CHNRI criteria)**. CHNRI- Child Health and Nutrition Research Initiative

We shared the initial review of the literature with 20 experts. The list of chosen experts included five leading basic scientists, five international public health researchers, five international policy makers and five representatives of the pharmaceutical companies. The 20 experts were chosen based on their excellent track record in child health research (but were not specifically involved with RSV disease research). We initially offered participation to the 20 experts with the greatest impact of publications in their area of expertise over the past 5 years (for basic researchers and international public health researchers), or for being affiliated to the largest pharmaceutical company in terms of vaccination programme or international agency in terms of their annual budget. For those who declined to participate (4 experts - about 20% - mainly due to conflicting arrangements/travel), replacements were found using the same criteria: for basic scientists and public health researchers we used Web of Knowledge and “ALRI” as search subject and limited time period to 2001-2008. This gave us a larger number of papers, which we sorted according to number of citations received. Then, we went down the ranks and invited the corresponding authors of the studies that were most relevant to the topic of our expert panel. The policy makers and industry representatives accepted our invitation on the condition of anonymity, due to sensitive nature of their involvement in such exercises. About half of the experts were either affiliated to institutions in developing countries or had previous experience of working in developing country settings. The experts met during September 7-13, 2009 in Dubrovnik, Croatia, to conduct the 2nd stage of CHNRI expert opinion exercise. The process of second-stage CHNRI is shown in Figure 2. All invited experts discussed the evidence provided in CHNRI stage I, and then answered questions from CHNRI framework (Supplementary table S2 in additional file 1). Their answers could have been “Yes” (1 point), “No” (0 points), “Neither Yes nor No” (0.5 points) or “Don’t know” (blank). Their “collective optimism” towards each criterion was documented on a scale from 0 to 100%. The interpretation of this metric for each criterion is straightforward: it is calculated as the number of points that each evaluated type of emerging RSV vaccine received from 20 experts (based on their responses to questions from CHNRI framework), divided by the maximum possible number of points (if all answers from all experts are “Yes“)[8-12].

## Results

**Figure 3:**
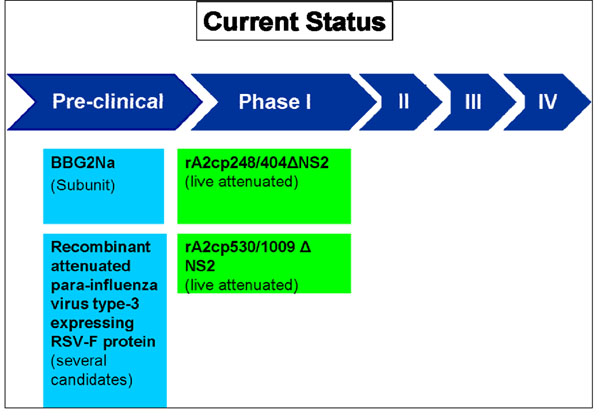
**The current status of the research into RSV vaccines for active immunization.** RSV- respiratory syncytial virus

**Figure 4:**
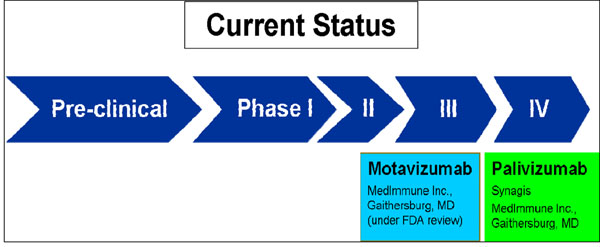
**The current status of the research into immunotherapy against RSV.** RSV- respiratory syncytial virus

We identified 70 articles and product monographs for inclusion. Currently several products are in development phase, most of which have completed phase I and II clinical trials (Figure 3 and Figure 4).

### Answerabilty - is the science behind the research viable?

The first RSV vaccine (a formalin inactivated whole virus preparation) developed over 40 years ago was associated with increased disease severity in the vaccine recipients compared to RSV infected controls [19]. Since then although there have been numerous reports (especially over the past two decades) of an effective vaccine being “just round the corner”, such a vaccine has been as elusive as ever.

#### Active immunization

The challenge currently facing live RSV vaccine developers is the appropriate balance between attenuation and immunogenicity [20]. This is a key challenge to overcome for RSV particularly as the virus primarily affects infants in the first 6 months of life [1]. The immune system at this age is immature and infants have a diminished B-cell response to infection which is an obstacle to achieving high titres of antibody [21]. Young infants also exhibit low T helper cells and inefficient antigen presentation. The poor response is accentuated by the effect of maternal antibodies, which have shown to suppress build-up of high serum neutralising antibody in response to immunization [22]. Live RSV vaccine shedding is not influenced by maternal antibody but is much greater in the naive child than in adults and older children with prior infection – a strong argument that there is substantial immunity to RSV. Thus there is only a narrow window between sufficient attenuation and effective immunogenicity.

Though there are shared epitopes, a potential vaccine may need to target two subtypes (A and B) further complicating the development process [23]. RSV has two proteins – F and G – which trigger the antibody response. Protein F and the central core of protein G remain constant in both strains and thus are major targets for subunit vaccines. G protein variability leads to the two antigenically distinguishable strains.

There is currently optimism regarding using reverse genetics technology to produce attenuated, genetically engineered live vaccines as a potential solution to several challenges faced in RSV vaccine development. The technique allows site directed mutations or gene deletions into the viral genome [24]. Several mutations can be introduced into the genome by this process to create a combination that achieves optimal levels of attenuation and immunogenicity. Current live vaccines under trial use gene deletion of protein NS2, which is known to prevent initiation of an innate immune response to viral infections [25]. Alternatively, chimeric vaccines can be created with a backbone of an attenuated virus other than RSV expressing immunogenic RSV proteins. This deletion has produced vaccines that appear to be sufficiently attenuated in infants and are thus a promising route [26].

**Figure 5:**
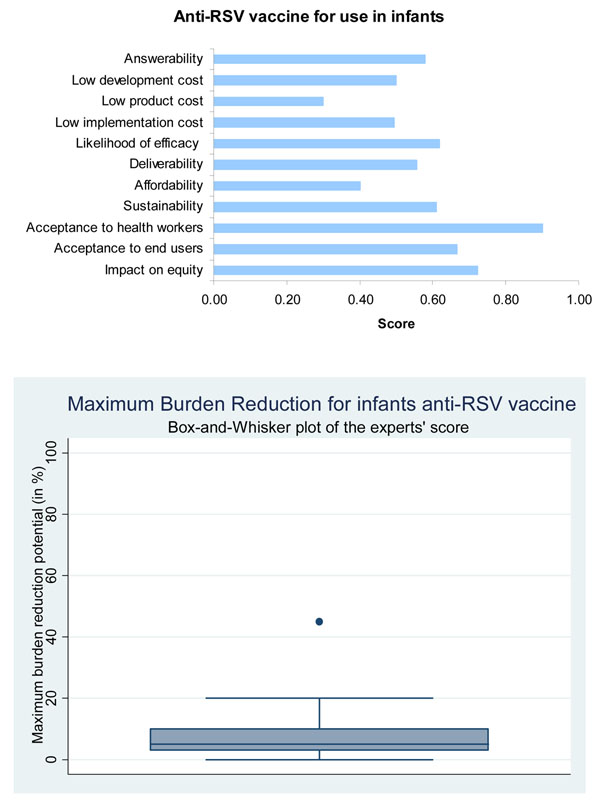
**The results of Stage II of the CHNRI process – an expert opinion exercise assessing the potential usefulness of investment in vaccines for active immunization of infants against RSV**. CHNRI- Child Health and Nutrition Research Initiative

Finding a suitable animal model for preclinical trials has been difficult due to host range restriction of RSV and the difficulty in mimicking the young age of the target population [21]. Most animal models are more resistant to the virus than young infants; thus the vaccine appears to be safer than it may be in the target population. The pace of development of novel vaccines is limited by the need for progressive clinical trials in adults and then in sero-positive children before it is deemed safe for trials in sero-negative infants. There is also a lack of a full understanding of the role played by the host immune system in the pathogenesis of natural RSV disease and in prediction of adverse reactions to vaccinations [21,27]. These challenges have prevented development of a vaccine for active immunization against RSV in infants below the age of 6 months. Nevertheless, there are currently several candidates at various stages of clinical trials and scientists are now hopeful of achieving in the near future a vaccine that is sufficiently attenuated and yet immunogenic and protective in young infants. The panel of experts expressed moderate levels of optimism (score around 60%) concerning the ability of vaccines for active immunization against RSV to satisfy the criterion of answerability (Figure 5).

#### Maternal immunization

**Figure 6:**
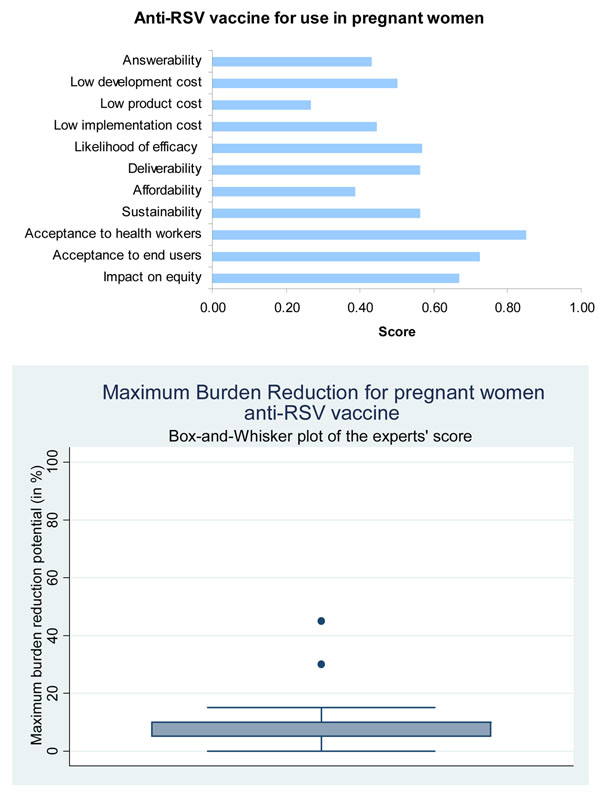
**The results of Stage II CHNRI process – an expert opinion exercise assessing the potential usefulness of investment in vaccines for immunization of pregnant women with vaccines against RSV**. CHNRI- Child Health and Nutrition Research Initiative

Maternal immunization aims to vaccinate women during late pregnancy in order to provide increased passive immunity to infants by antibodies transferred from placenta and breast feeding. Antibodies are transferred from mother to foetus by active transport after 32 weeks gestation [28]. It has been shown that high levels of maternal antibody protect babies from severe RSV related disease and hospitalisation in the first year of life [29,30]. This is particularly promising as a successful candidate maternal vaccine would protect infants aged less than 6 months who form the bulk of the disease burden and for whom it is proving to be difficult to develop active immunization. Only one candidate of a purified fusion protein (PFP) subunit vaccine has thus far entered clinical trials [31,32]. This approach is strengthened by the ability of higher titre monoclonal antibodies to protect infants when given prophylactically in the post-partum period. Glezen and colleagues have demonstrated that the protection against RSV infection in early infancy is correlated with maternal antibody [33]. Active transport of maternal antibodies only occurs during the last trimester and may not be effective in case of premature babies (at particular risk of serious RSV related illness). Further trials using PFP subunit vaccines has since been discontinued since there are concerns about the safety of subunit RSV vaccines [34]. Presented with this evidence, the panel of experts expressed a low level of optimism (score around 40%) concerning the ability of vaccines for maternal immunization against RSV to satisfy the criterion of answerability (Figure 6).

#### Passive immunization

**Figure 7:**
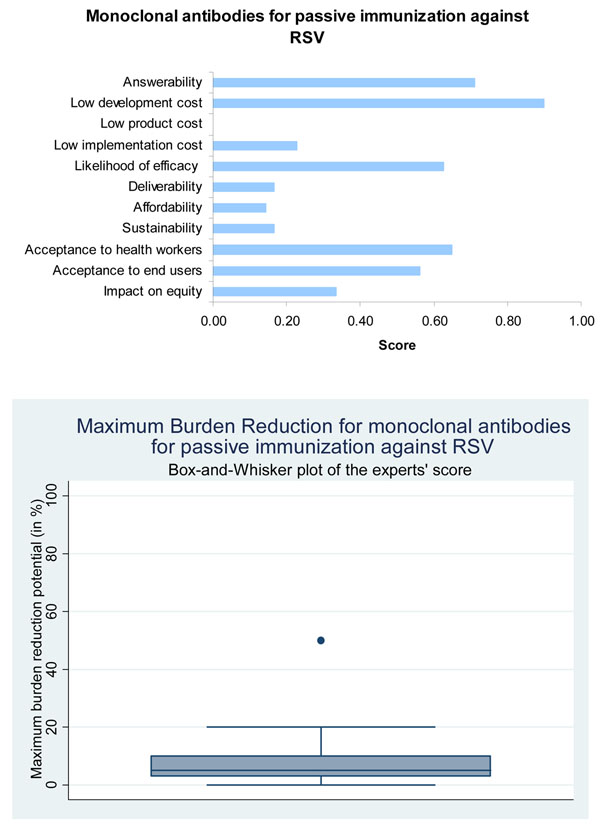
**The results of Stage II CHNRI process – an expert opinion exercise assessing the potential usefulness of investment in monoclonal antibodies for passive immunization of infants against RSV**. CHNRI- Child Health and Nutrition Research Initiative

Current vaccines for passive immunization against RSV deliver protection against active disease to infants at high risk during the peak RSV season. These interventions raise serum neutralizing antibody. There have been two products in the market; RSV immune globulin (RespiGam; MedImmune Inc., Gaithersburg, MD) containing high-titre human polyclonal RSV antibodies [35] and Palivizumab (Synagis; MedImmune Inc.) a humanized murine monoclonal antibody against RSV [36]. The more recent Palivizumab is now widely used as it has fewer adverse effects. In countries where Palivizumab is currently used; it is largely only approved for the prevention of severe RSV disease in premature infants, those with bronchopulmonary dysplasia or haemodynamically unstable chronic heart failure. A more potent derivative of Palivizumab – Motavizumab –has been evaluated and shows increasing efficacy against medically attended LRI but was non inferior for RSV hospitalization [37]. It is now awaiting US FDA approval [38]. Based on these evidence, the panel of experts expressed moderate to high levels of optimism (score around 70%) regarding the ability of monoclonal antibodies to satisfy the criterion of answerability (Figure 7).

### Efficacy - the impact of the vaccines under ideal conditions

#### Active immunization

The efficacy results of the various candidate vaccines for active immunization against RSV are summarized in Table 2. Immunization with RSV vaccine is unlikely to prevent RSV infection altogether [39]. Natural infection in an infant does not mount a robust enough immune response to prevent subsequent infection. A study in Texas shows that 83% of those infected in the first year of life were again infected in the second and 46% in their third year, showing that risk of infection only reduces after the second infection and second year [40]. It is thus hypothesised that a new candidate is likely to need multiple doses at frequent intervals to achieve adequate immunity against RSV infection [41], making it difficult to have a successful immunization programme in low-income countries. Thus any vaccine against RSV which would eventually be licensed may only be able to provide protection against severe ALRI, hospitalisation and death which account for the greatest disease burden. It is also likely that these vaccines could prevent sequelae and bacterial super-infection as a result of RSV infection.

All of the live vaccines are being designed for intranasal delivery using the model of the cold-adapted influenza vaccine, Flumist®. It is hoped that this will increase its efficacy by enhancing mucosal immunity as this is believed to play an important role in RSV infection. Despite several trials of subunit vaccines, none have been conducted on young sero-negative children. Such trials are unlikely to be seen in the near future mainly because of our incomplete understanding of the events leading to the enhanced disease seen with the Formalin-inactivated vaccine [34] and the fact that development of PFP, (the most promising candidate), and other subunit vaccines have been discontinued due to almost uniformly low immunogenecity.

A recombinant attenuated para-influenza type-3 candidate incorporating RSV protein F has also been created using reverse genetics technology and aims to protect against both viruses [42,43]. A candidate vaccine was recently shown to be safe in seropositive children though with inadequate immunogenicity [44]. There are a host of other candidates using other viruses as vectors such as adenovirus, Sendai virus, Newcastle disease virus, vaccinia virus and Venezuelan equine encephalitis virus which are beyond the scope of this paper [45-53]. Presented with these evidence, the panel of experts expressed moderate levels of optimism (score around 60%) regarding the ability of vaccines for active immunization against RSV to satisfy this criterion (Figure 5).

**Table 2:** Efficacy results of candidate vaccines for active immunization against respiratory syncytial virus

Class	Vaccine structure	Clinical trial phase	Results
**Live attenuated**[74]	rA2cp248/404ΔNS2 rA2cp530/1009 Δ NS2	I	- Not infectious in adults - Well tolerated, no symptomatic illness - Infected 50% and 20% sero-negative infants respectively at a dose of 10^5^ pfu
**Live attenuated**[24]	rA2*cpts*248/404/1030/ Δ SH	I	- only candidate with a demonstrated safety profile - 44% vaccinated infants had detectable antibodies after 2 doses of 5.3log_10_ pfu
**b/hPIV3/RSVF2**[44]	Recombinant attenuated para-influenza virus type-3 expressing RSV-F protein	I	- tested in 120 1-9 year old sero-positive children. - acceptable safety profile - minimally immunogenic
**Subunit**[32,75,76]	Purified F Protein - PFP 1 and PFP 2	Discontinued after phase I/ II	- Pilot study shows significant antibody titres in children with CF - Safe and immunogenic in 12-48 month old sero-positive children
**Subunit**[75,76]	PFP 3	Discontinued after phase II	- Double blinded controlled multi-centre study in CF children - Safe and immunogenic but no reduction in LRTI
**Subunit**[75,76]	BBG2Na	Animal models	- Safe and immunogenic in adult mice. - Phase III trials in adult volunteers stopped due to unexpected adverse effects^24^

#### Maternal immunization

The subunit vaccine using Purified Fusion Protein-2 was not immunogenic enough in mothers and only low antibody titres were achieved thus necessitating the need for a more potent candidate vaccine [31]. Furthermore, research into PFP candidates has since been discontinued. The panel of experts however expressed moderate levels of optimism (score around 60 percent) regarding efficacy of the vaccine in case one such were to be developed (Figure 6).

#### Passive immunization

Passive immunization against RSV with monoclonal antibodies is highly efficacious. The results of the recent trials using monoclonal antibodies are summarized in Table 3. Here too the experts expressed moderate levels of optimism (score around 60%) regarding efficacy of this intervention (Figure 7).

**Table 3:** Efficacy results of candidate vaccines for passive immunization against respiratory syncytial virus

Class	Vaccine structure	Clinical trial phase	Results
**Human polyclonal**			
[77,78]	RSV IVIG	Passed clinical trials	**-** 40.7% relative reduction in hospitalization compared to placebo **-** 63.4% relative reduction in hospitalization compared to placebo
**Humanized monoclonal Ig**[36]	Palivizumab	Passed clinical trials	**-** 54.7% relative reduction in hospitalisation compared to placebo
**Humanized monoclonal Ig**[37]	Motavizumab	III	- 50% relative reduction in medically attended LRI compared to Palivizumab. - 26% relative reduction in hospitalisation compared to Palivizumab.

### Effectiveness- maximum burden reduction potential

Nair and colleagues estimated that in the year 2005, 33.8 million new episodes of RSV-associated ALRI occurred globally in children aged less than 5 years, of which 3.4 million were severe enough to result in hospitalisation [1]. Ninety six percent of these episodes were in developing countries. They also estimated that in the year 2005, roughly 53,000 to 199,000 children younger than 5 years of age died from RSV associated ALRI, with 99% of these deaths occurring in developing countries.

Developing an effective vaccine for active immunization against RSV would result in a significant reduction of disease burden from RSV infections. However, at present it is not possible to precisely quantify of the maximum reduction of disease burden using the RSV vaccines without any information on the vaccine effectiveness. None of the vaccine candidates have passed phase III trials. The potential for a RSV vaccine for herd immunity also remains to be seen.

Though effectiveness of maternal immunization against RSV in the general population cannot be elicited at this stage, there are potential barriers to attaining a high degree of effectiveness in low-income countries. High levels of malaria in pregnant women are worrying as this has been shown to impede active placental transport in the case of maternal immunization against tetanus. In The Gambia, there was a 58% reduction in the transfer of trans-placental antibody against RSV in association with placental malaria [54].

Several trials have been carried out to assess effectiveness of Palivizumab across high-income countries such as USA, Canada, France and Netherlands [55]. A study in the USA including 2095 children showed hospitalization rates of 2.9% in infants on Palivizumab. In a Canadian study with 480 infants hospitalisation rates were half of that quoted in the Impact-RSV study [36]. Though these studies have consistently reinforced the safety and high effectiveness of Palivizumab, none of these have been conducted in low and middle-income countries. The panel was of the opinion that candidates for all three interventions are likely to have low levels of maximum impact on overall pneumonia disease burden (Figures 5, 6 and 7).

### Cost of development, product and implementation and affordability

In the case of candidate vaccines for active immunization of infants against RSV, and also the candidate vaccines targeting pregnant women, the experts expressed very low levels of optimism for low product cost, affordability and low cost of development. The group also evaluated immunoprophylaxis against RSV using monoclonal antibodies and expressed no optimism at all towards low product cost; very low levels of optimism regarding affordability and low implementation cost, but high levels of optimism regarding low development cost (Figures 5,6,7). Clearly, cost of the product and its implementation in developing country settings has been the major concern of the expert panel related to all emerging interventions against RSV.

### Deliverability, sustainability and acceptability

#### Active immunization

Live attenuated vaccines – currently the most promising candidates – are being developed with intranasal delivery. Along with a likely increase in effectiveness, this would also make delivery easier due to minimal training needs and a potential saving in total cost due to this. Such a delivery method would obviate transmission of blood borne infections such as Hepatitis B and HIV associated with poor needle handling in low-income countries. With candidates being at very early stages of trials, there is little indication of where-if at all-it may fit into the expanded programme on immunization (EPI) schedule [56]. However, a potential limitation of a likely candidate is the need for multiple doses. This may reduce uptake depending on the number of doses and the time interval between each, as it may not complement the current EPI schedule. Additionally, there are no data on interactions of candidate vaccines with others in the EPI schedule [57]. It is also hugely concerning that unlike Flumist®, all live attenuated candidates thus far have required storage facilities below -70°C which would not fit with current cold chains in most countries and is not at all feasible in low-income countries [23]. It is hoped that this obstacle would be overcome as the vaccine progresses through further clinical trial stages. Finally, the safety of live RSV vaccines, in relationship to development of recurrent wheezing and asthma later on is a theoretical concern but needs to be studied before it is widely used. The panel expressed moderate levels of optimism (score around 60 percent) on the criteria of deliverability, sustainability and acceptability to end users of these vaccines for active immunization against RSV (Figure 5). However, they expressed high level of optimism (score over 80%) on the acceptability of these vaccines to the health workers.

As candidate vaccines have not yet reached phase IV clinical trials, there are no cost estimates for these new interventions. However, being a new vaccine, initial prices are likely to be high especially with a high demand for the vaccine in high-income countries. A way of making an emerging intervention more deliverable in low and middle-income countries could be with differential pricing of the product [58]. This works by licensing a vaccine with the agreement of setting lower and more affordable prices in low and middle-income countries. The model has potential to work well in the case of RSV as there is a great demand for a RSV vaccine in the developed countries, which would thus be able to heavily subsidise the cost in the developing countries. Since majority of the disease burden due to RSV and influenza occur in very young children (in the first two years of life), and both diseases have strong seasonal pattern of transmission and the lower respiratory infection associated with both are known to progress rapidly, extrapolation of cost effectiveness using analyses for an influenza vaccine may be useful. Salo and colleagues demonstrated that investing 1.1 million Euros on vaccinating children with an influenza vaccine between 6 months and 3 years, reduced medical costs by 2.8 million Euros, thereby resulting in a cost-saving 1.7 million Euros [59]. This is particularly promising as incidence rates were underestimated and showed potential savings with a vaccine efficacy as low as 60%. Since the burden of disease with RSV is higher than that from influenza one could expect greater savings [60]. Additionally, benefits of potential herd immunity must be considered. However, this particular analysis was carried out in Finland and thus cannot be generalised globally. The experts however, expressed low levels of optimism (score less than 60 percent) regarding the ability to develop the vaccine at a low cost (Figure 5).

#### Maternal immunization

Health care utilization indicators suggest that an effective delivery system is in place for maternal immunization in many middle and low-income countries. Even in countries with low hospital delivery levels, a majority of women still attend antenatal care at least once. Greenwood points out that more than 50% of women in 24 of 28 African countries surveyed were found to attend antenatal clinics on four or more occasions [61]. In addition, in many malaria endemic countries, greater attendance is being encouraged through prevention programs in order to deliver prophylactic treatment. The high coverage achieved by maternal immunization programs against tetanus is also particularly encouraging as neonatal tetanus is a particular problem of extremely poor communities [62]. However, since vaccines for maternal immunization are still in early stages of development, there is as yet no indication of storage requirements for these vaccines. The experts expressed moderate levels of optimism (score around 60 percent) on the criteria of deliverability and sustainability of these vaccines (Figure 6). They however were more optimistic (score around 80 percent) on the acceptability of these vaccines to the end users and health workers. The panel expressed concern (score around 50 percent) about the ability to develop these vaccines at a low cost.

#### Passive immunization

Delivery of Palivizumab requires monthly injections for five months through the RSV season; this may prove difficult in low-income countries for several reasons. In the tropical and sub-tropical regions the seasonality of RSV is not very clear as in temperate regions [63,64]. Unlike in the US, the administration of Palivizumab in developing countries would most likely rely on the availability of hospital services which would make delivery difficult in resource poor settings which constitute the bulk of the disease burden. Administration of Palivizumab is only recommended in high-risk patients. Since this decision is based on clinical judgement, it is likely to be a limiting factor in low and middle-income countries where there is shortfall in health manpower. Palivizumab needs to be stored at 2 to 8° C which makes it suitable for utilizing existing cold chain facilities available under EPI [65]. Immunoprophylaxis with Palivizumab has primarily been in developed countries due to the high cost associated with it. Most economic analyses show that Palivizumab use is not cost-effective though some studies show greater cost-efficacy specifically for use in premature infants [66]. Economic analyses for the use of Palivizumab in Argentina demonstrated a cost of $15 358 per avoided hospitalization while the same was at a cost of $34 840 in an Aborigine community [67,68]. Studies in Malaysia show that a significantly better outcome was achieved for patients with RSV bronchiolitis in hospitals where better intensive care expertise and resource was available [69]. Many critics have argued that in countries where health resources are scarce, money may be better channelled into improving intensive care units than investing in an expensive new intervention. Since monoclonal antibodies have already been developed, the experts were highly optimistic (score around 90 percent) that they could be made available to low and middle income countries at a low cost (Figure 7). However, they were not optimistic (score around 20 percent) regarding the deliverability and sustainability of these interventions. They were however moderately optimistic (score around 60 percent) regarding the acceptability of these products to the end users and health workers.

### Impact on equity

Though RSV affects young children across the world, 96 percent of the episodes occur in developing countries [1]. Thus an equitable coverage program needs to reach a vast proportion of the global poor. Evaluation of current immunization and treatment programs show that uptake of a new initiative is faster and more efficient in rich populations between and within countries. This is called inverse equity as the global poor are exposed to higher degrees of disease, yet effective interventions fail to reach them [70]. In many countries where several interventions are being promoted simultaneously (instead of encouraging universal coverage), these provide cumulative benefit to the rich and increase health inequalities. An evaluation of coverage of several interventions in 54 priority countries showed an average coverage of over 50% with less than 30% coverage in the poorest communities [71].

This disparity is less apparent in immunization programs than with some other interventions. Yet, in 50 low and middle-income countries average coverage for full immunization was 62% in the richest 20% and a mere 38% in the poorest 20% [72]. These evaluations highlight a large and growing equity gap which is precipitated by the addition of new interventions. While these interventions may be effective and succeed in reducing disease burden, a greater proportion of deaths could be prevented by aiming for universal coverage with fewer interventions. Thus a new RSV vaccine is only likely to be equitable if a novel delivery system that aims to target poor populations is adopted [73]. The panel was moderately optimistic (score around 70 percent) about the ability of the vaccines for active and maternal immunization to have an impact on equity (Figures 5, 6, and 7). However, they were not optimistic (score around 40 percent) about the ability of monoclonal antibodies to satisfy this criterion.

## Discussion

RSV is the most common cause of ALRI in children and an important cause of child mortality with a high disease burden in low and middle-income countries. The literature review summarized in this paper presents evidence required for making an informed decision on the research priority that should be given to emerging interventions against RSV. The scores for active and passive immunization of infants and pregnant women with interventions against the set criteria represent the collective optimism of a panel of experts drawn from varying technical backgrounds and affiliations. Although there are currently no vaccines to protect against the virus, significant progress is being made for active immunization, with live attenuated preparations looking most promising. Recent research has increased hope for a successful vaccine for infants as young as 2 months. However, there have been no trials in low and middle-income countries which are essential to assess their impact in these areas where disease burden is highest. Pharmaceutical companies would need to seriously consider undertaking future clinical trials in developing countries without which no progress in reducing global childhood mortality associated with RSV can be anticipated.

In the case of candidate vaccines for active immunization of infants against RSV, the experts expressed low levels of optimism for cost of product, affordability and low cost of development and implementation; moderate levels of optimism regarding the criteria of answerability, likelihood of efficacy, deliverability, sustainability and acceptance to end users for the interventions; and high levels of optimism regarding impact on equity and acceptance to health workers. While considering the candidate vaccines targeting pregnant women, the panel expressed low levels of optimism for cost of product, affordability, low cost of development and implementation, and even answerability; moderate levels of optimism for likelihood of efficacy, deliverability, sustainability and impact on equity; high levels of optimism regarding acceptance to end users and health workers. The group also evaluated immunoprophylaxis against RSV using monoclonal antibodies and expressed no optimism at all towards low product cost; low levels of optimism regarding deliverability, affordability, sustainability, cost of implementation and the impact on equity; moderate levels of optimism against the criteria of answerability, likelihood of efficacy, acceptance to end-users and health workers; and high levels of optimism regarding low development cost. As far as the vaccines against RSV are concerned the challenge would be to develop a low cost, immunogenic yet safe vaccine which can be either given to infants younger than two months of age or develop one which can be given to pregnant women in their last trimester.

This is the first time such an exercise has been conducted with the aim of predicting the future impact of emerging vaccines. The CHNRI methodology was primarily designed to evaluate existing interventions and competing investment priorities for health research. Although we used the CHNRI criteria, we modified it by including systematic review of available literature and not involving all stakeholders (e.g. end-users and health workers). The scores reported in this paper express the collective opinion of a panel of 20 experts. While there is always an element of error while predicting impact of interventions which do not exist and have no clinical trial data to support them (especially efficacy and maximum disease burden reduction potential), we feel that the results would be reproducible with another panel in a different setting.

## Conclusions

To summarize, while it is not only important that investments are made in researching new vaccines, adequate emphasis must be made and resources allocated for proper distribution of the vaccine. While vaccines for active immunization of infants appear to be the most promising, the search for a candidate vaccine which is immunogenic yet sufficiently attenuated is not yet over. It looks unlikely that maternal immunization would provide sufficient protection to young infants. And while monoclonal antibodies have proven to be effective in providing protection to high-risk infants, high costs and need for hospitalisation for delivery severely limit their generalisability. As more and more countries introduce vaccines against Streptococcus pneumoniae and Haemophilus influenzae type B in their EPI, and coverage of these vaccines increases, the burden of disease due to bacterial pneumonias will inevitably decrease, thus further increasing the relative importance of viral causes. Moreover, as most bacterial pneumonias are secondary to viral ALRI, introduction of an effective vaccine against RSV will have a compounded effect on the overall morbidity and mortality due to childhood pneumonia.

## Competing interests

EAFS has in the past received consultancy, grants, and honoraria for lectures from MedImmune and grants from Roche; however, no grants or honoraria were received for work included in this study. PFW has in the past received grant funding and honoraria from Sanofi-Aventis, Wyeth, MedImmune and Merck; however, no grants or honoraria were received for work included in this study. HN, VRV, ET, LZ, TH, IR and HC declare that they have no competing interests.

## Authors’ contributions

HN participated in the design of the study, literature review, data collection, data analysis, data interpretation and prepared the initial draft of the manuscript. VRV participated in the design of the study, led the literature review, contributed to data collection and preparation of the initial draft of the manuscript. ET participated in design of the study, data collection, statistical analysis, data interpretation and critically reviewed the manuscript. LZ participated in the design of the study, data collection, data collection and critically reviewed the manuscript. TH, EAFS and PW contributed to data interpretation and critical review of the manuscript. IR and HC conceived of the study, participated in literature review, data collection, data interpretation, and critically reviewed drafts of the manuscript. All authors read and approved the final manuscript.

## Supplementary Material

Additional file 1 Supplementary tables 1 and 2. Click here for file
