# Supplementary material for: An evaluation of the emerging interventions against Respiratory Syncytial Virus (RSV)-associated acute lower respiratory infections in children
Source: BMC Public Health. 2011 Apr 13;11(Suppl 3):S30. doi: 10.1186/1471-2458-11-S3-S30 (PMC3231904; doi:10.1186/1471-2458-11-S3-S30)
Supplement: Additional file 1 — Supplementary tables 1 and 2. [file 1471-2458-11-S3-S30-S1.doc]

# Supplementary material for “An evaluation of the emerging interventions against Respiratory Syncytial Virus associated acute lower respiratory infections in children”

# Supplementary Table 1: Details of search strategy for identifying studies reporting novel interventions against RSV

### Vaccine for active or passive immunisation against RSV

| **Number** | **Searches** | **Results** |
| --- | --- | --- |
| 1 | Respiratory Syncytial Virus Infections/ | 3463 |
| 2 | RSV.tw | 6125 |
| 3 | respiratory syncytial virus.tw. | 7187 |
| 3 | 1 or 2 or 3 | 9847 |
| 4 | vaccin$.ti,ab. | 164063 |
| 5 | immuni$.ti,ab | 175117 |
| 6 | viral vaccines/ or exp respiratory syncytial virus vaccines/ | 17871 |
| 7 | immunization/ or exp immunization, passive/ | 63144 |
| 8 | (passive adj3 vaccin$).ti,ab | 238 |
| 9 | 4 or 5 or 6 or 7 or 8 | 324317 |
| 10 | child/ or exp infant/ | 1668391 |
| 11 | child$.ti,ab | 777689 |
| 12 | infan$.ti,ab | 270245 |
| 13 | 10 or 11 or 12 | 1890809 |
| 14 | 3 and 9 and 13 | 872 |

### Vaccines for maternal immunisation against RSV

| **Number** | **Searches** | **Results** |
| --- | --- | --- |
| 1 | Respiratory Syncytial Virus Infections/ | 3463 |
| 2 | RSV.tw | 6125 |
| 3 | respiratory syncytial virus.tw. | 7187 |
| 3 | 1 or 2 or 3 | 9847 |
| 4 | vaccin$.ti,ab. | 164063 |
| 5 | immuni$.ti,ab | 175117 |
| 6 | maternal immuni$.ti,ab | 532 |
| 7 | (mother$ adj3 immuni$).ti,ab | 529 |
| 8 | immun$ pregnan$.ti,ab | 554 |
| 9 | pregnan$ vaccin$.ti,ab | 28 |
| 10 | 4 or 5 or 6 or 7 or 8 or 9 | 290776 |
| 11 | 3 and 10 | 1701 |

### Deliverability and equity

| Number | Searches | Results |
| --- | --- | --- |
| 1 | vaccin$.ti,ab. | 164063 |
| 2 | immuni$.ti,ab | 175117 |
| 3 | 1 or 2 | 290776 |
| 4 | Respiratory Syncytial Virus Infections/ | 3463 |
| 5 | RSV.tw | 6125 |
| 6 | respiratory syncytial virus.tw. | 7187 |
| 7 | 1 or 2 or 3 | 9847 |
| 8 | deliver$.mp. | 346581 |
| 9 | deliver$.ti,ab. | 277021 |
| 10 | EPI.mp. | 10067 |
| 11 | EPI.ti,ab. | 8825 |
| 12 | exp immunization/ or exp immunization, passive/ or exp immunization schedule/ or exp immunization, secondary/ or exp immunotherapy, active/ or exp vaccination/ or exp mass immunization/ | 117192 |
| 13 | cost$.ti,ab. | 248051 |
| 14 | "health care facilities, manpower, and services"/ or "health care economics and organizations"/ or "costs and cost analysis"/ or health planning/ | 57339 |
| 15 | 8 or 9 or 10 or 11 or 12 or 13 or 14 | 728492 |
| 16 | 3 and 7 and 15 | 495 |

### Disease Burden

| **Number** | **Searches** | **Results** |
| --- | --- | --- |
| 1 | Respiratory Syncytial Virus Infections/ | 3463 |
| 2 | RSV.tw | 6125 |
| 3 | respiratory syncytial virus.tw. | 7187 |
| 4 | 1 or 2 or 3 | 9847 |
| 5 | (disease adj3 burden).ti,ab. | 6170 |
| 8 | 4 and 5 | 70 |

# Supplementary Table 2: Questions used in the Phase II CHNRI process

(Please answer: “1” = YES; “0” = NO; “0.5” = I can’t tell; “blank = I don’t know)

**ANSWERABILITY**

- Do we have a sufficient research and development capacity to make the intervention available on the market by 2020?
- Do we have a sufficient level of funding support to make the intervention available on the market by 2020?
- Would you say that it is likely that the remaining technical hurdles can be overcome to make the intervention available on the market by 2020?

**COST TO DEVELOPMENT**

- Would you say that in order to get from current stage of development to commercial availability of each emerging intervention below we would need to still invest < 1 billion US$?
- Would you say that in order to get from current stage of development to commercial availability of each emerging intervention below we would need to still invest < 500 million US$?
- Would you say that in order to get from current stage of development to commercial availability of each emerging intervention below we would need to still invest < 100 million US$?

**COST OF PRODUCT, AFFORDABILITY AND COST OF IMPLEMENTATION**

- Is it likely to be a low-cost intervention (i.e. <3.50 US$ per unit?)
- Is achievement of a near-universal coverage likely to be affordable to most developing countries?
- Can we use the existing delivery mechanisms without major modifications (e.g. training, infrastructure)?

**EFFICACY AND EFFECTIVENESS**

Please assess the likelihood (0%-100%) that adequately powered randomized controlled trials of the intervention (RSV vaccine), conducted in developing countries, would consistently show statistically significant reduction in cause-specific mortality from each of the four causes of child death- pneumonia, meningitis, neonatal sepsis and influenza.

**MAXIMUM POTENTIAL FOR DISEASE BURDEN REDUCTION**

Please predict, for each of the 4 causes of child death (pneumonia, meningitis, neonatal sepsis and influenza), the proportion of deaths in children under five years of age due to that cause that could be averted if the complete coverage with the emerging intervention (RSV vaccine) could be achieved?

**DELIVERABILITY AND SUSTAINABILITY**

Taking into account (i) the infrastructure and resources required to deliver emerging interventions listed below (e.g. human resources, health facilities, communication and transport infrastructure); (ii) the resources likely to be available to implement the emerging interventions at the time of introduction; (iii) overall capacity of the governments (e.g. adequacy of government regulation, monitoring and enforcement; governmental intersectoral coordination), and (iv) internal and external partnership required for delivery of interventions (e.g. partnership with civil society and external donor agencies), would you say that the emerging interventions would be:

- Deliverable* at the time of introduction?
- Sustainable for at least 10 years at the time of introduction?

**ACCEPTABILITY TO HEALTH WORKERS, END USERS AND EFFECT ON EQUITY**

Taking into account the overall context, intervention complexity, health workers’ behaviour and the end-user population at the time of introduction, please specify:

- Would health workers be likely to comply with implementation guidelines?
- Would end-users be likely to fully accept the intervention?
- Would you say that the proposed intervention has the overall potential to improve equity after 10 years following the introduction?
